# Supplementary material for: IGF-1-enhanced miR-513a-5p signaling desensitizes glioma cells to temozolomide by targeting the NEDD4L-inhibited Wnt/β-catenin pathway
Source: PLoS One. 2019 Dec 5;14(12):e0225913. doi: 10.1371/journal.pone.0225913 (PMC6894868; doi:10.1371/journal.pone.0225913)
Supplement: S1 Fig — (A) Total patients (n = 519). (B) The group with lower miR-513a expression levels (n = 260). By using the median cutoff for miR-513a expression levels, the patients were divided into two groups. The correlation was calculated by Pearson's correlation analysis. (PDF) [file pone.0225913.s001.pdf]

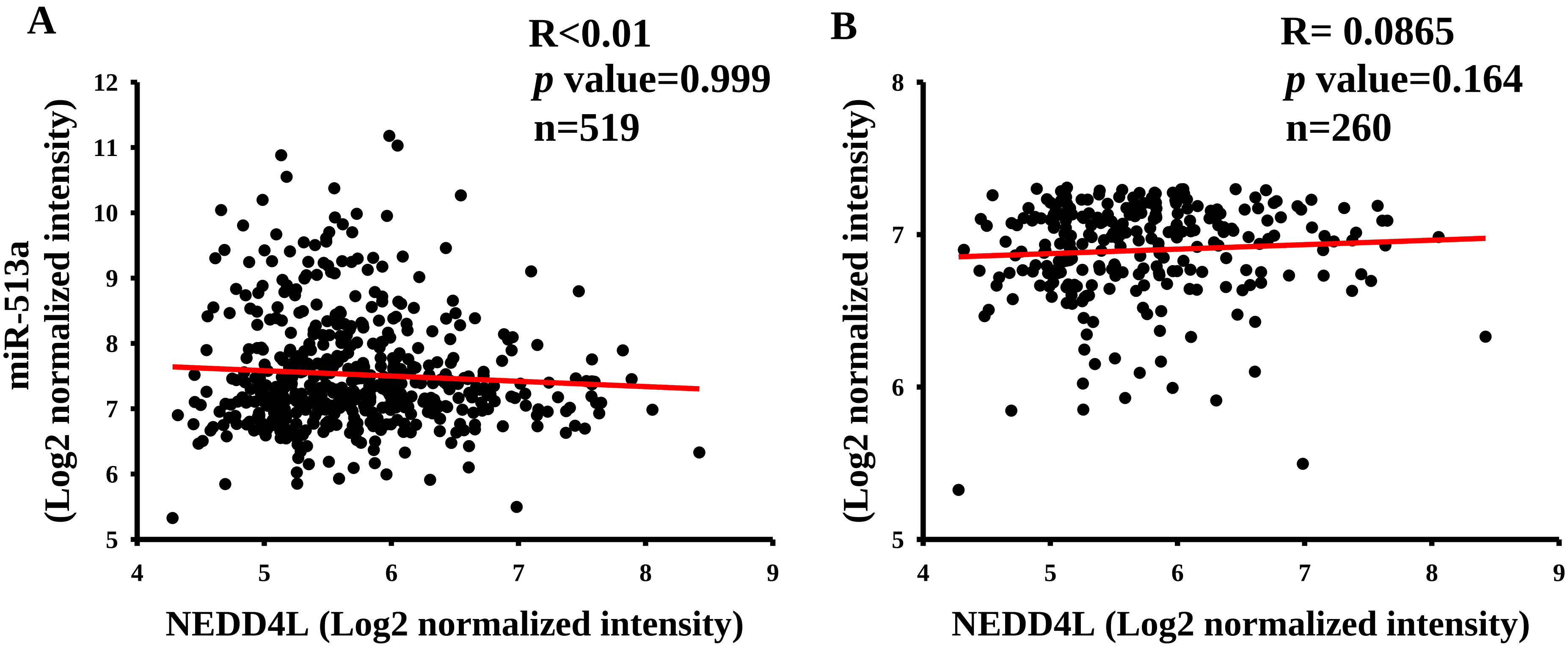

S1 Fig. The correlation between miR-513a and NEDD4L expression levels in TCGA microarray data. (A) Total patients (n=519). (B) The group with lower miR-513a expression levels (n=260). By using the median cutoff for miR-513a expression levels, the patients were divided into two groups. The correlation was calculated by *Pearson's correlation analysis.*
