# Supplementary material for: IGF-1-enhanced miR-513a-5p signaling desensitizes glioma cells to temozolomide by targeting the NEDD4L-inhibited Wnt/β-catenin pathway
Source: PLoS One. 2019 Dec 5;14(12):e0225913. doi: 10.1371/journal.pone.0225913 (PMC6894868; doi:10.1371/journal.pone.0225913)

S2 Fig. Raw data of immunoblotting assays.

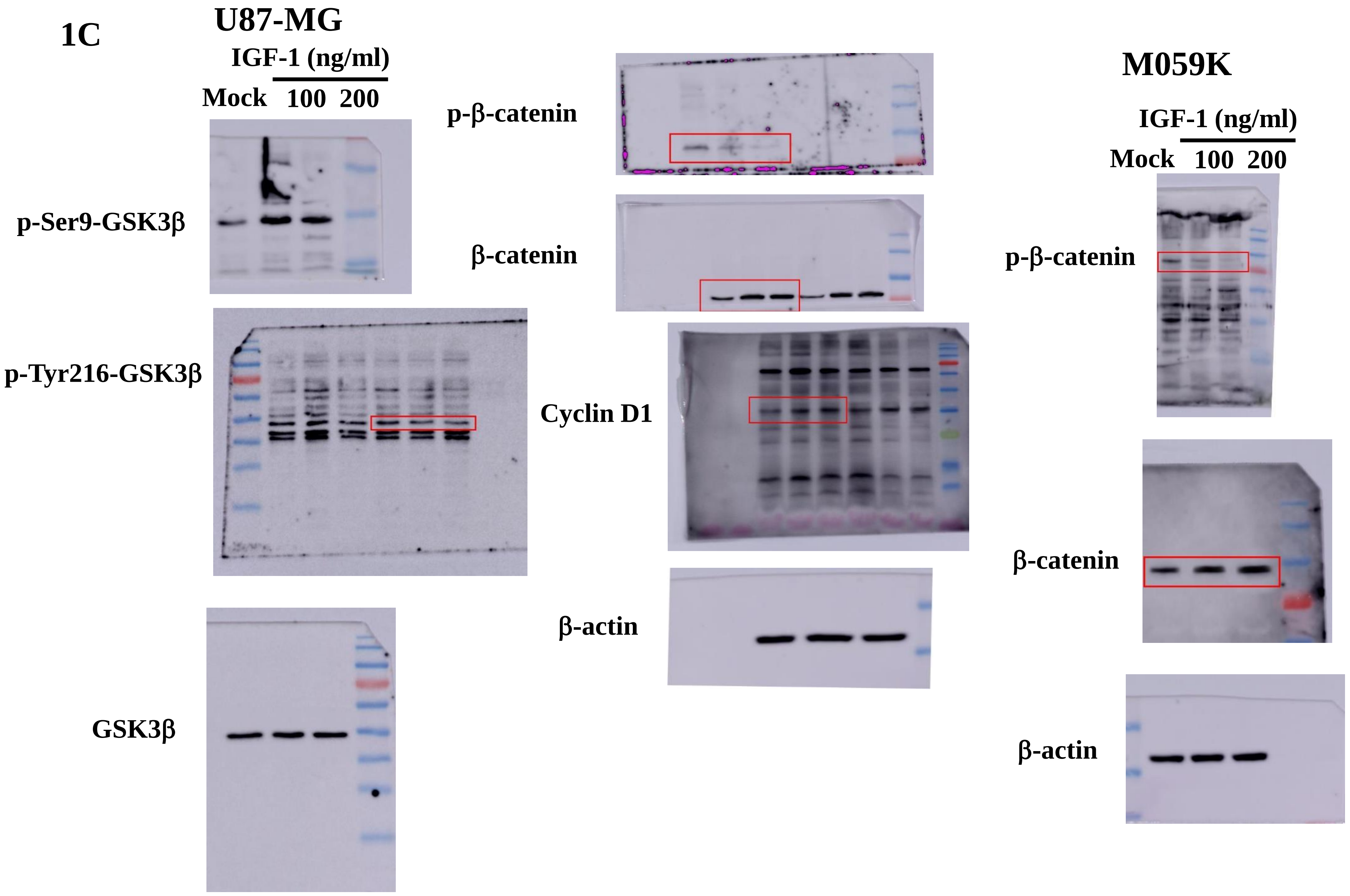

Continued S2 Fig.

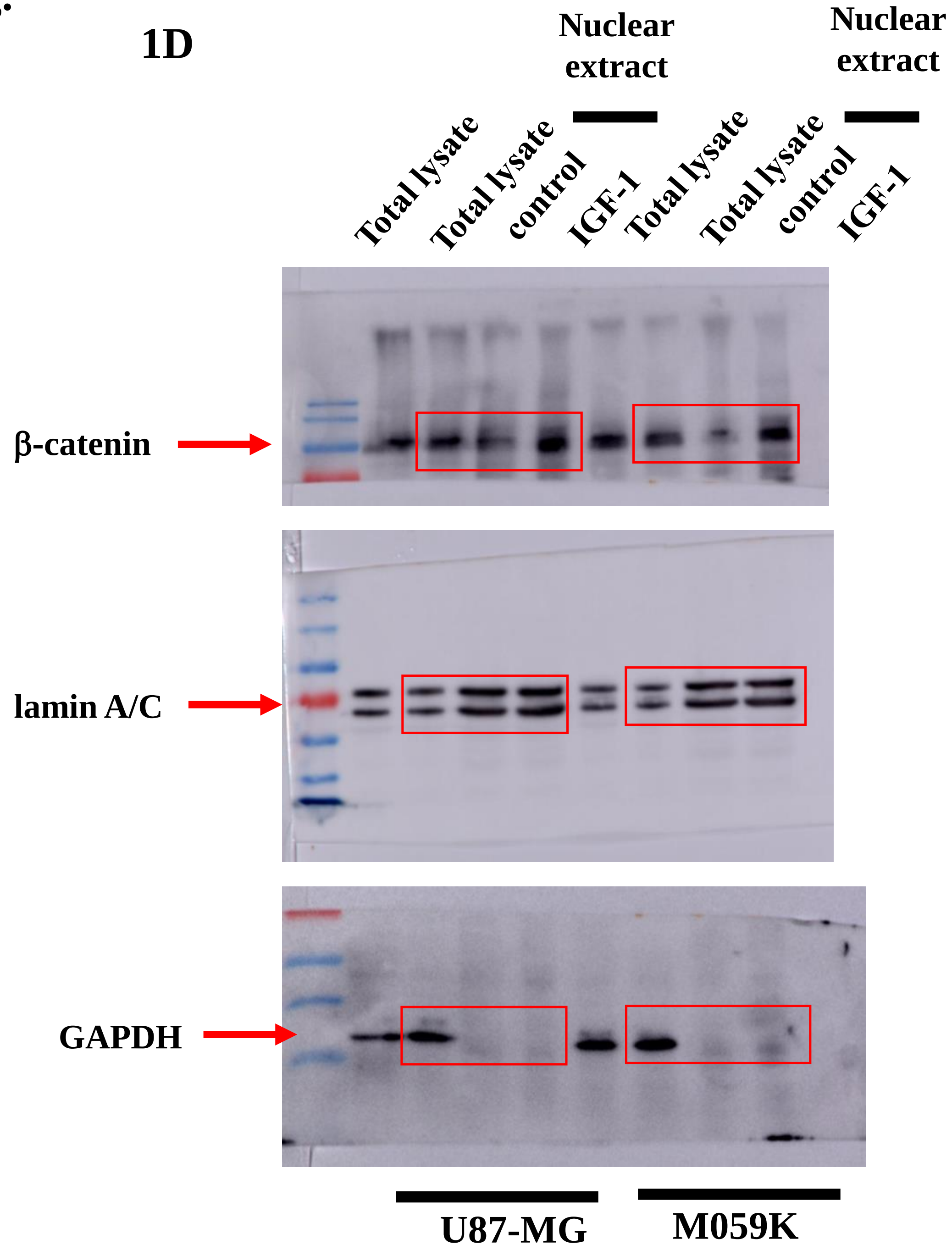

Continued S2 Fig.

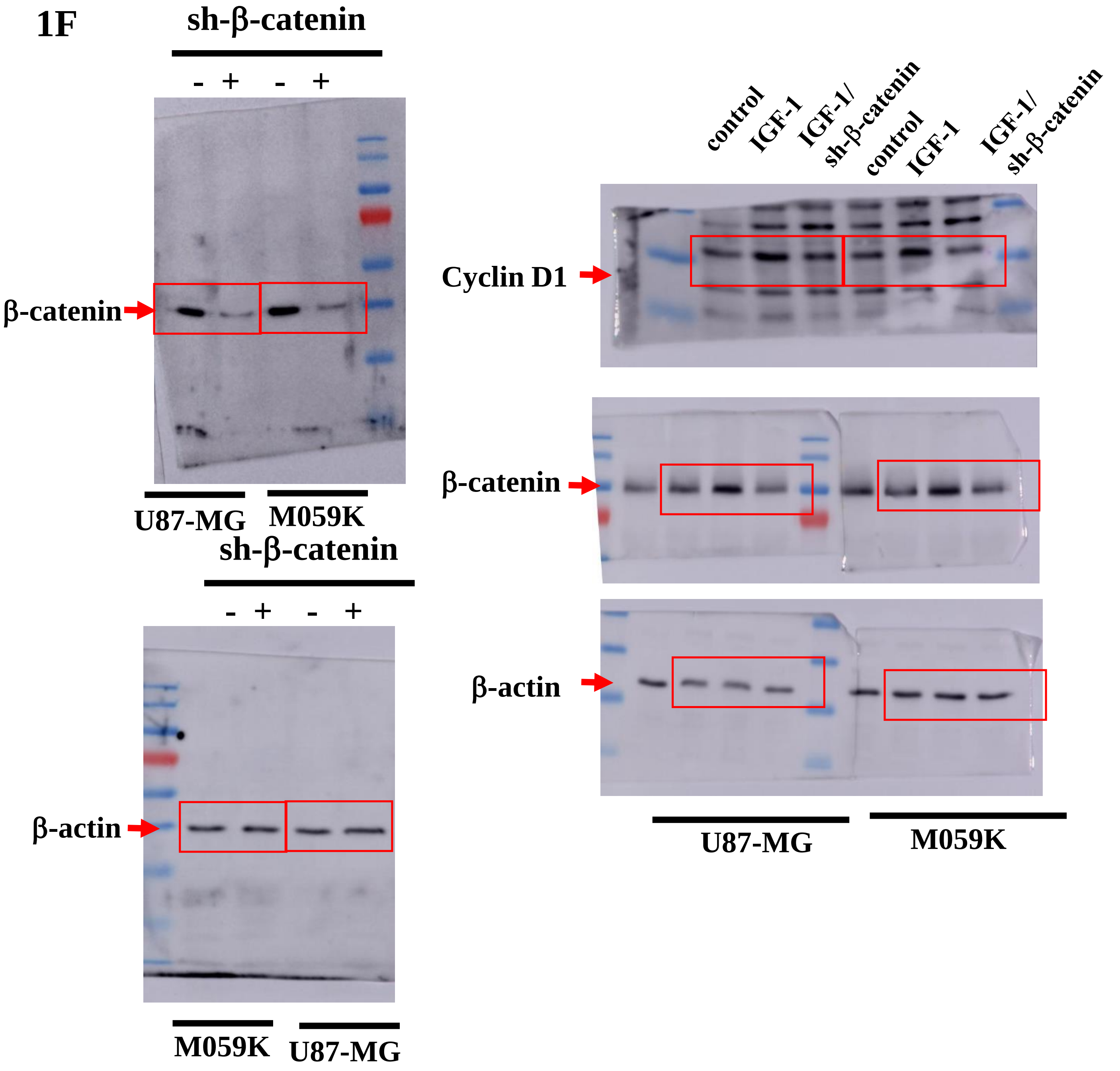

Continued S2 Fig.

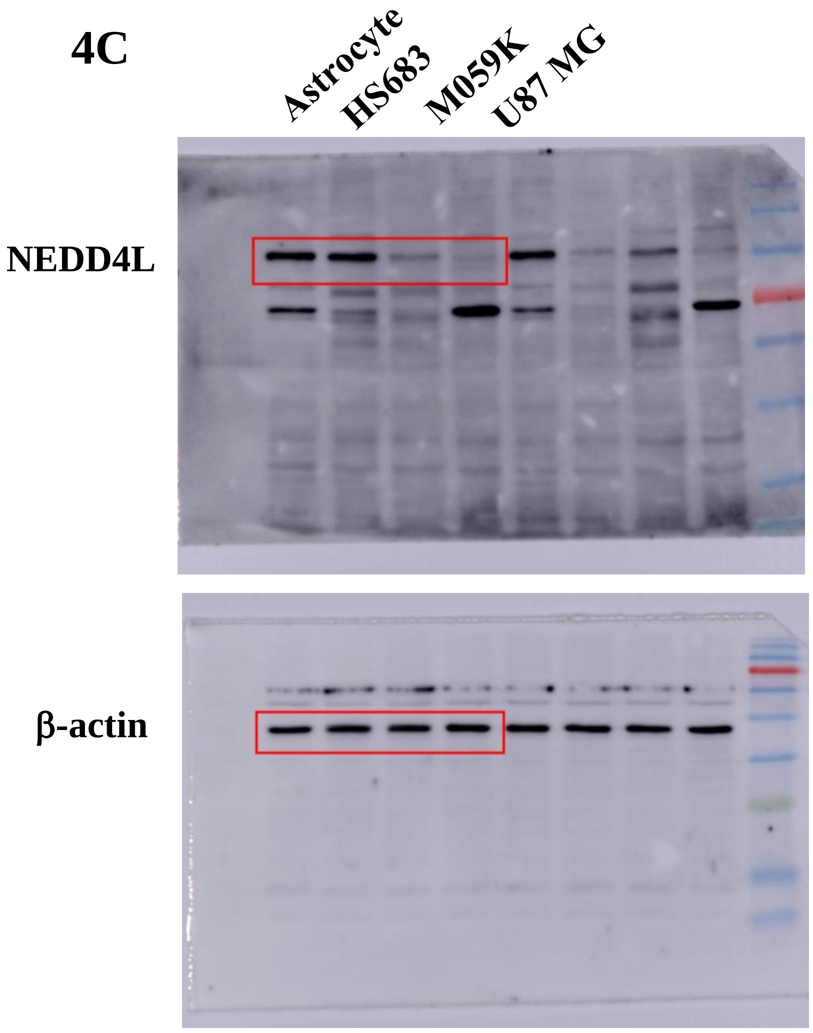

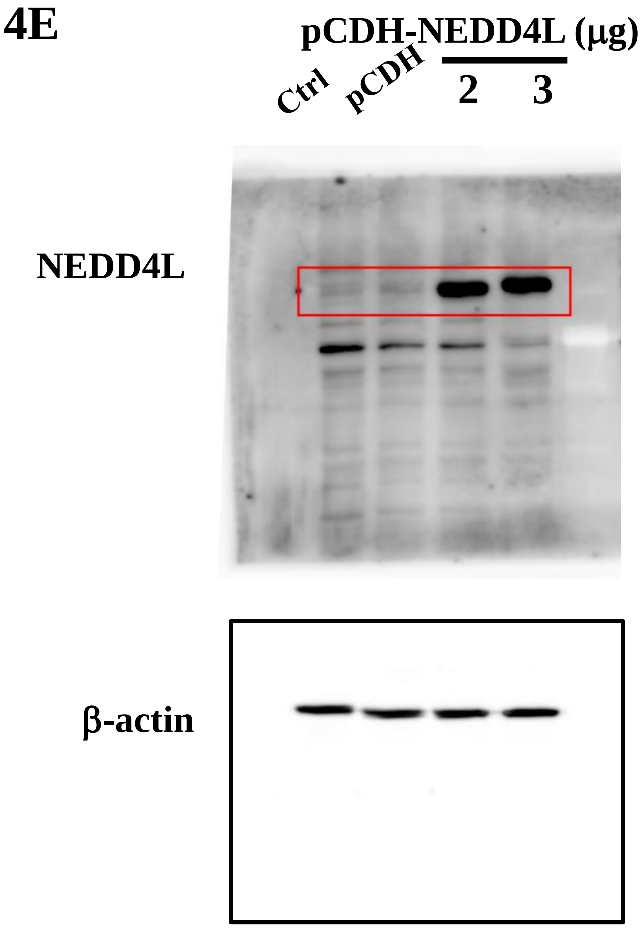

Continued S2 Fig.

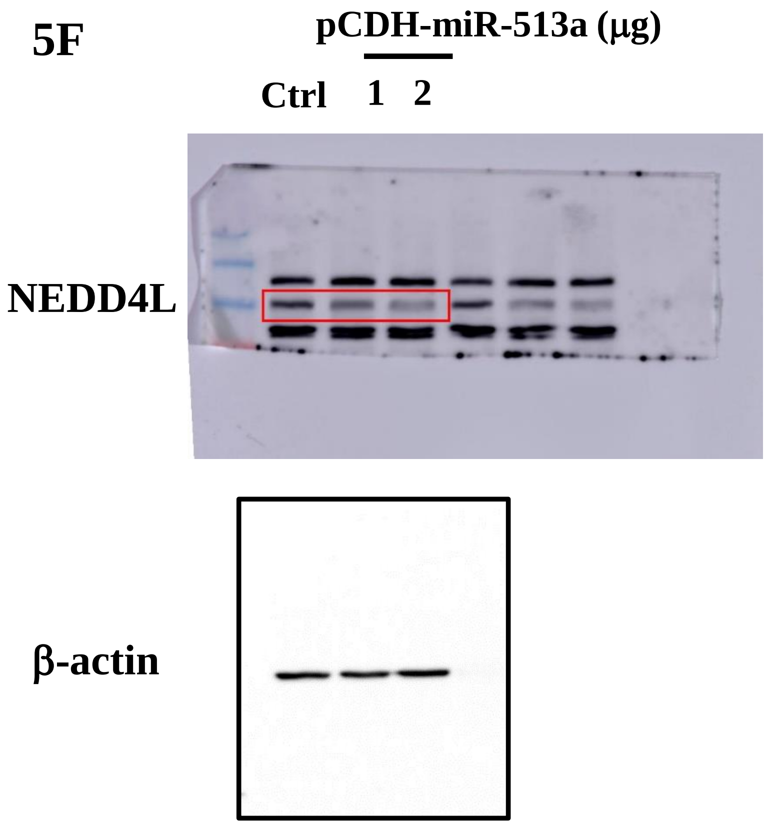

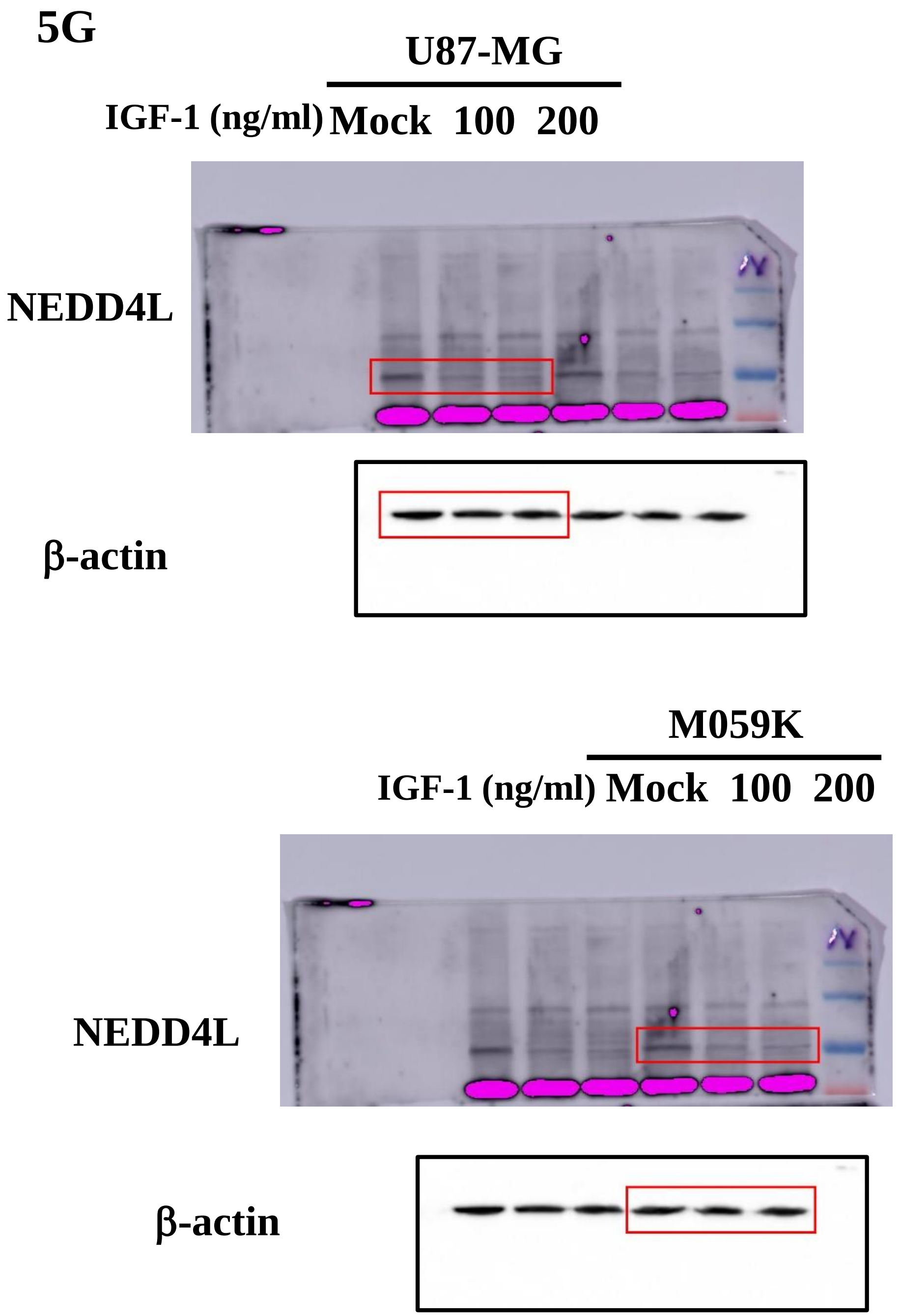

Continued S2 Fig.

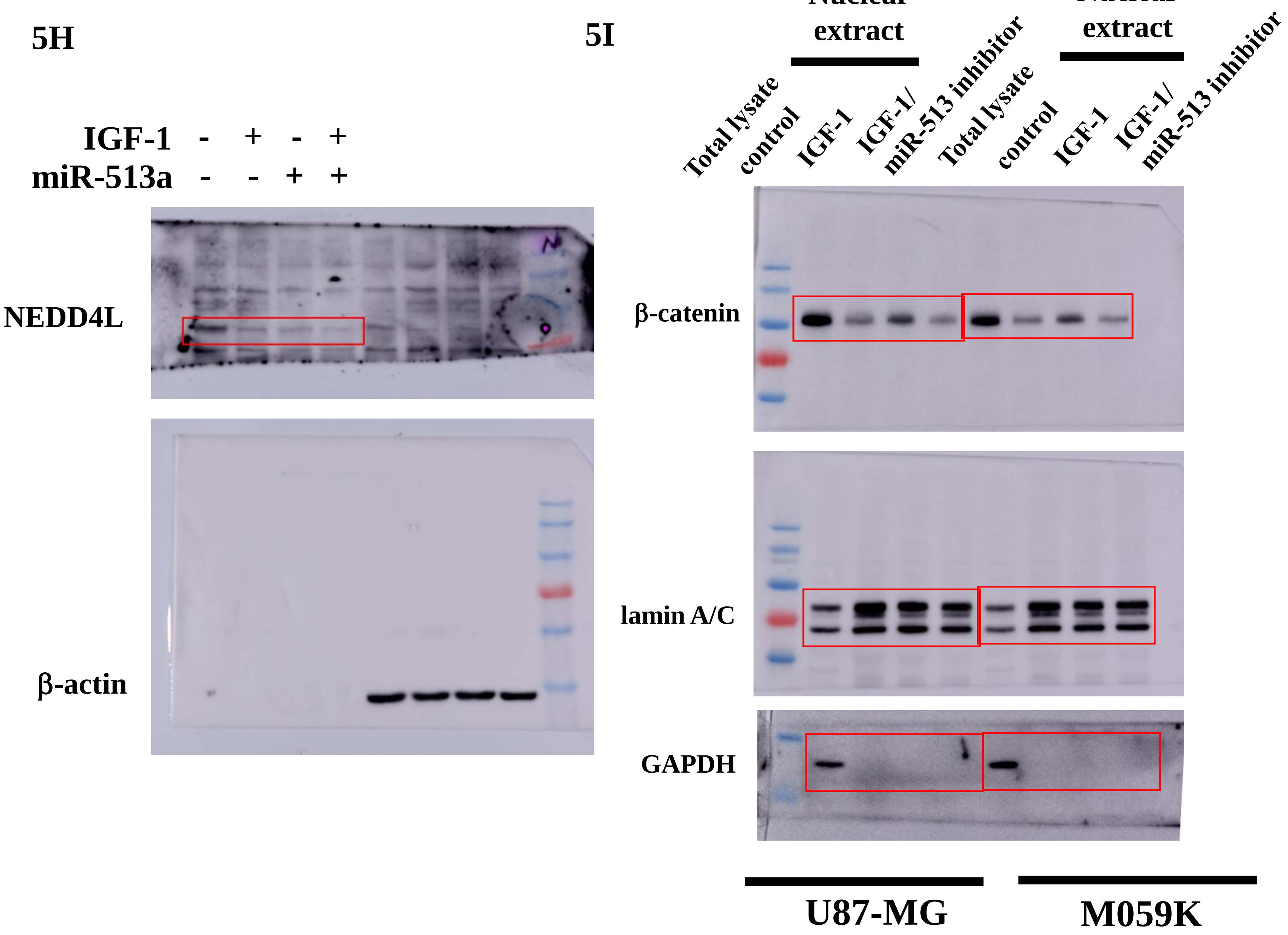

Continued S2 Fig.

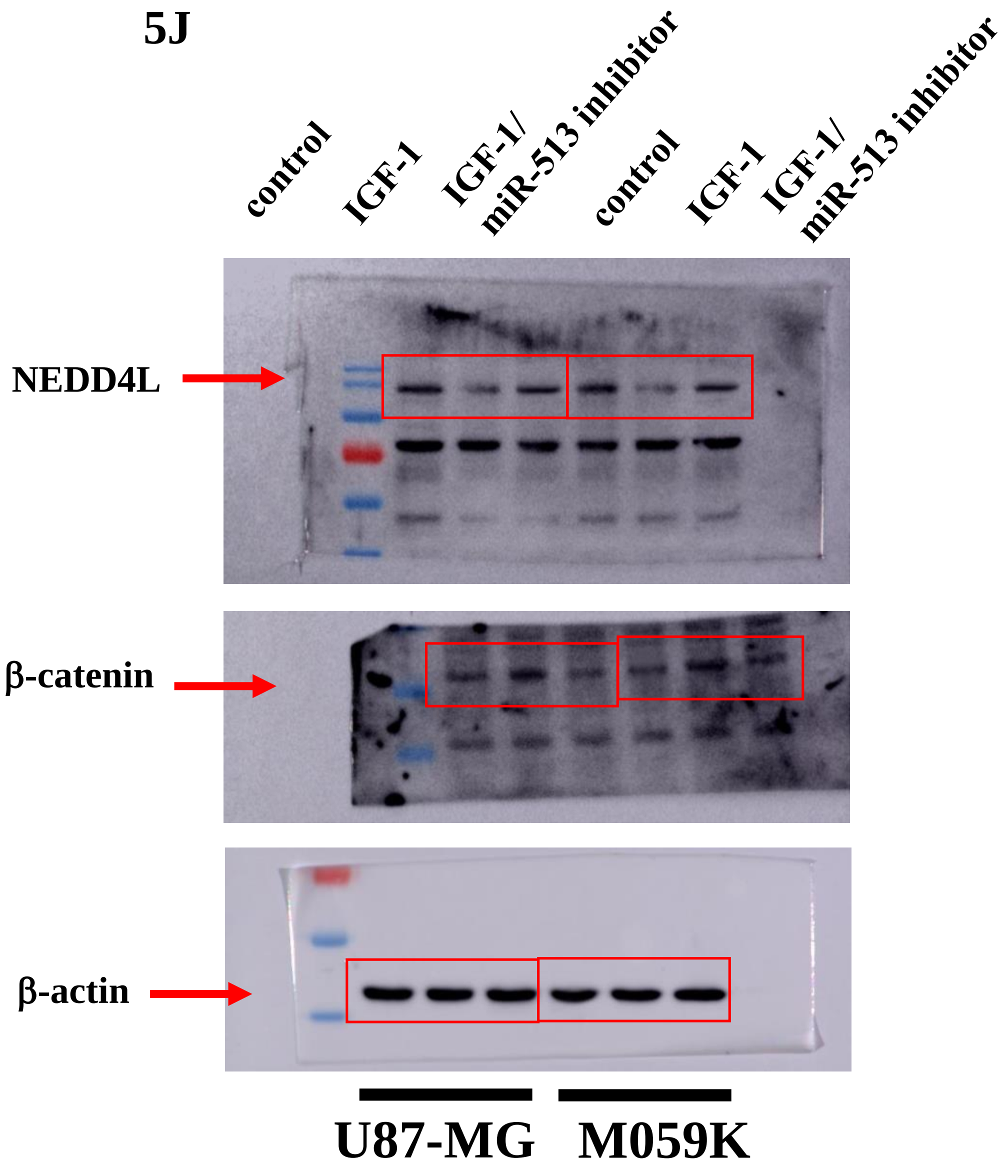

Continued S2 Fig.

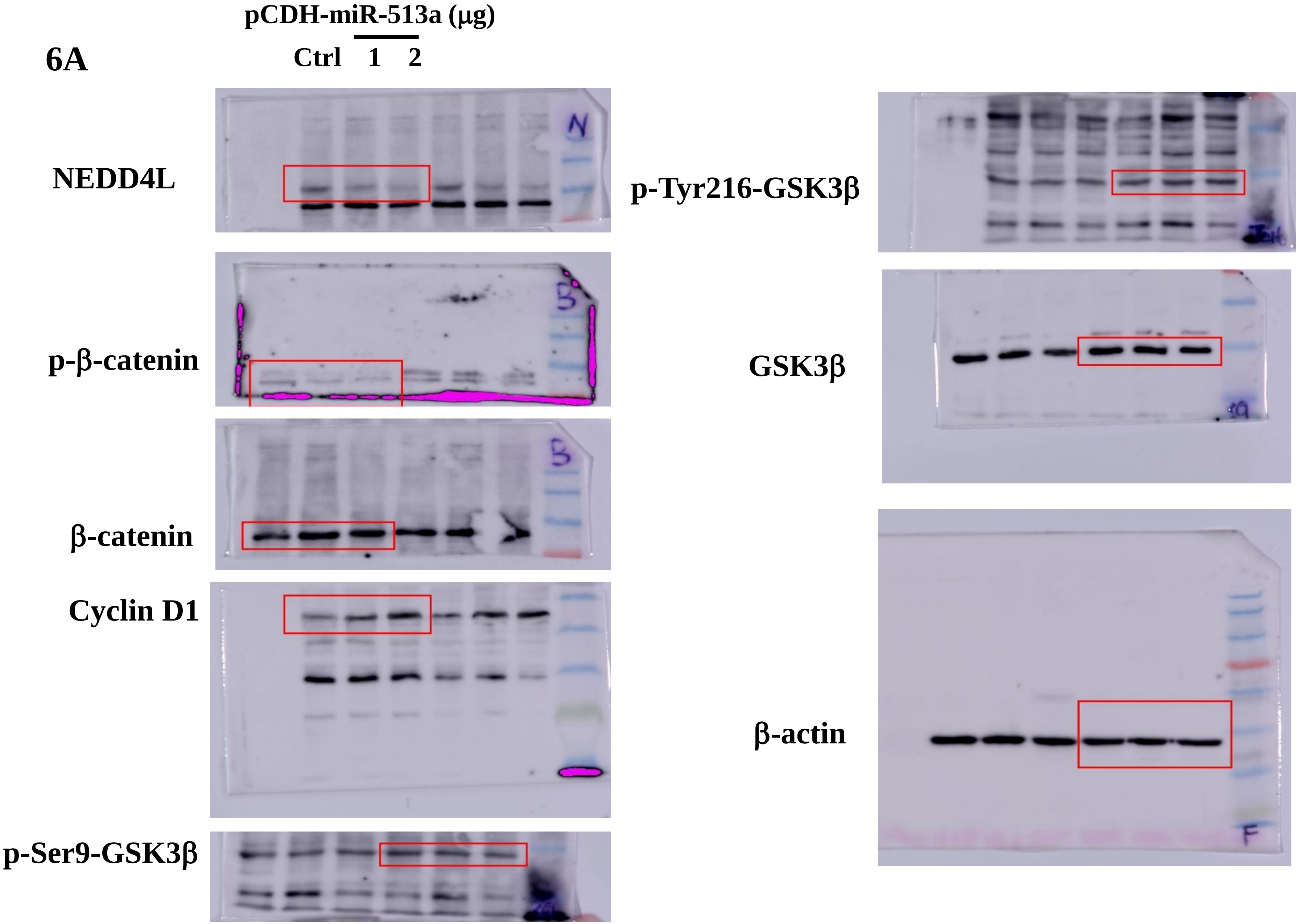

Continued S2 Fig.

6B

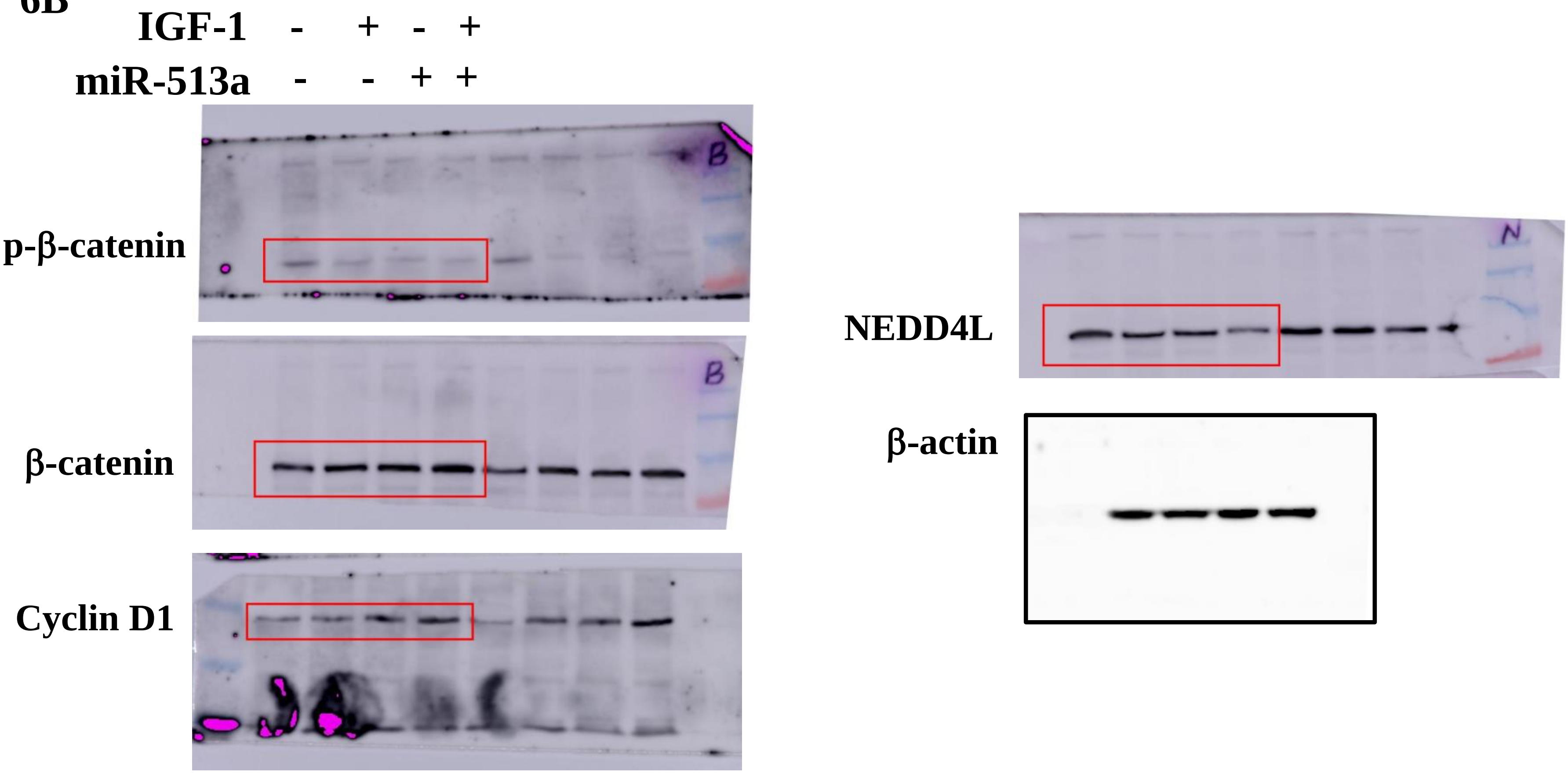

Continued S2 Fig.

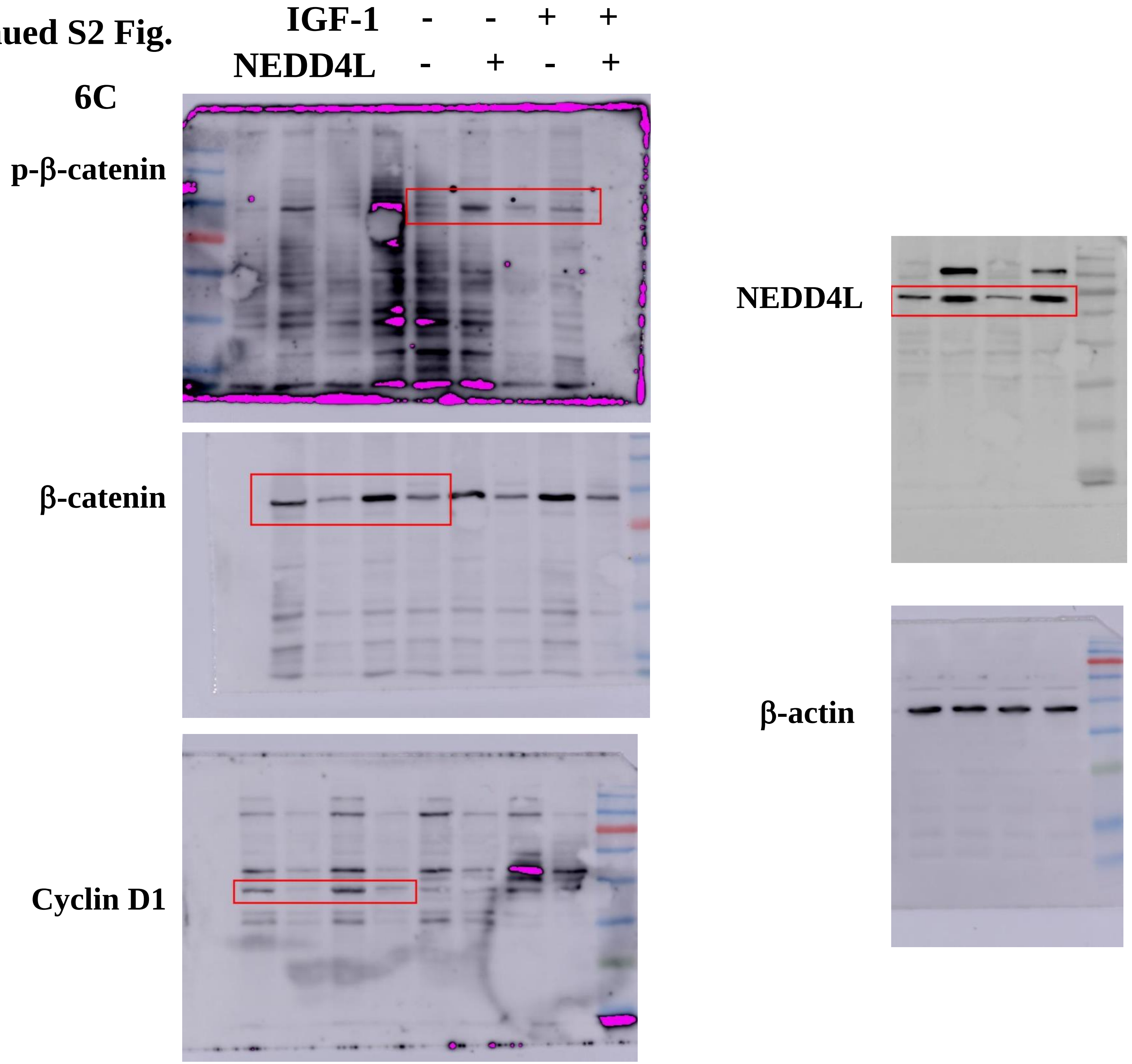

Supplement: S2 Fig — (PDF) [file pone.0225913.s002.pdf]
