# Supplementary material for: IGF-1-enhanced miR-513a-5p signaling desensitizes glioma cells to temozolomide by targeting the NEDD4L-inhibited Wnt/β-catenin pathway
Source: PLoS One. 2019 Dec 5;14(12):e0225913. doi: 10.1371/journal.pone.0225913 (PMC6894868; doi:10.1371/journal.pone.0225913)
Supplement: S1 Table — (PDF) [file pone.0225913.s003.pdf]

S1 Table. Primer list

Primer name	Sequence
**For NEDD4L gene cloning**	
NEDD-clon-F	ACTTCTAGAATGGAGCGACCCTATACATTTAAGG
NEDD-clon-R	ACTGCTAGCTTAATCCACCCCTTCAAATCC
**For miR-513a gene cloning**	
miR-513a-clon-F	CATAGAATTCCAAGTTGCATTGTCCCTTGGCATAAATC
miR-513a-clon-R	ATGCGGATCCAGGCACAAAAAGTTCCTTGAAGTGTAGC
**For NEDD4L 3'UTR cloning**	
NEDD-3UTR-F	TATCTCGAGTTGCGTGACTTTGTTCTTCCT
NEDD-3UTR-R	ATCTCTAGAAAGCGAAAGGATTGTTCTTCA
**For 3'UTR Mutagenesis**	
NEDD-3UMUT-F	TTTTCTCCAAGCTGTGTATCAGTCATTTCAACCCTTGGAGCC
NEDD-3UMUT-R	GGCTCCAAGGGTTGAAATGACTGATACACAGCTTGGAGAAAA
**For real-time PCR**	
NEDD4L-sybr-F	GACATGGAGCATGGATGGGAA
NEDD4L-sybr-R	GTTCGGCCTAAATTGTCCACT
GAPDH-sybr-F	GTG AAG GTC GGA GTC AAC
GAPDH-sybr-R	GTT GAG GTC AAT GAA GGG
