# Supplementary material for: IGF-1-enhanced miR-513a-5p signaling desensitizes glioma cells to temozolomide by targeting the NEDD4L-inhibited Wnt/β-catenin pathway
Source: PLoS One. 2019 Dec 5;14(12):e0225913. doi: 10.1371/journal.pone.0225913 (PMC6894868; doi:10.1371/journal.pone.0225913)
Supplement: S2 Table — (PDF) [file pone.0225913.s004.pdf]

S2 Table. List of IGF-1-upregulated miRNAs in U87 MG cells (log2 (Ratio) ≧ 0.58; adj. *p* value ≦ 0.05)

Name	Normalized Intensity		CV*		log2 (Ratio)	AveExpr**	P.Value	adj. *p* value***
	Control	IGF-1	Control	IGF-1				
hsa-miR-513a-5p	376.145	1511.695	0.07950005	0.11197648	2.004552805	9.555138345	0.000336	0.003783
hsa-miR-513b	130.5	509.375	0.08127664	0.05229293	1.966077907	8.008558435	0.000128	0.003168
hsa-miR-4299	139.5	442.7	0.10644618	0.07644484	1.668051795	7.95404887	0.000514	0.004352
hsa-miR-4672	82	220.89	0.05173952	0.02669777	1.430341331	7.071756508	8.81E-05	0.003044
hsa-miR-3687	94.5	225.86	0.0673435	0.16661747	1.248597178	7.184903447	0.003163	0.010677
hsa-miR-4492	1381.095	3272.415	0.04310444	0.0265153	1.244959979	11.05340639	9.67E-05	0.003044
hsa-miR-1247-3p	103	241.51	0.01373023	0.09697063	1.226107064	7.299486062	0.000572	0.004486
hsa-miR-30c-1-3p	456	1033.865	0.00620269	0.01963606	1.180816881	9.423284578	2.46E-05	0.002683
hsa-miR-4684-3p	86	193.01	0	0.03179984	1.165902215	7.009215862	4.62E-05	0.003044
hsa-miR-1275	2147.81	4812.195	0.01436067	0.07441219	1.161903232	11.64952789	0.000332	0.003783
hsa-miR-4508	1106.5	2457.22	0.01342001	0.0910207	1.148094235	10.68576989	0.000587	0.004486
hsa-miR-3714	110.5	244.735	0.05759241	0.11403969	1.143665478	7.358537992	0.001516	0.007438
hsa-miR-4753-5p	113.25	246.285	0.19043494	0.10746496	1.129840365	7.375087379	0.007534	0.018145
hsa-miR-4539	126	273.02	0	0.04672261	1.114795124	7.534677485	0.000113	0.003044
hsa-miR-6129	123	264.5	0.01149767	0.00267337	1.10465451	7.494794079	2.04E-05	0.002683
hsa-miR-4283	84.5	177.865	0.00836813	0.09433921	1.070567539	6.936137949	0.000789	0.005018
hsa-miR-30b-3p	163	338.365	0.06940925	0.17618895	1.044164045	7.869070482	0.006272	0.016003
hsa-miR-3934-5p	119.5	245	0.04142048	0.11544601	1.03156712	7.41603131	0.001845	0.008136
hsa-miR-513c-5p	77.5	158.525	0.00912396	0.07230532	1.030584094	6.791386427	0.000433	0.004097

hsa-miR-1321	119.5	239.075	0.02958606	0.01611934	1.000674716	7.400888386	8.20E-05	0.003044
hsa-miR-3945	256.5	510.9	0.02481076	0.04124444	0.993690361	8.499438141	0.000173	0.003423
hsa-miR-4417	93	183.77	0.03041319	0.05209896	0.981952693	7.029801471	0.000308	0.003783
hsa-miR-3667-5p	86.5	168.84	0.02452393	0.14373315	0.957611658	6.913217106	0.003808	0.012187
hsa-miR-652-5p	157.5	299.545	0.02244783	0.03750998	0.927095046	7.762573773	0.000173	0.003423
hsa-miR-575	99	187.825	0.01428499	0.08150599	0.92156225	7.090064142	0.000859	0.005253
hsa-miR-1273c	97	183.765	0.02915904	0.0520619	0.921133844	7.060173036	0.000365	0.003783
hsa-miR-1236-5p	331	623.505	0.02136274	0.02042485	0.913584051	8.827314814	8.73E-05	0.003044
hsa-miR-4531	80.5	151.56	0.04391968	0.06065181	0.912196398	6.786319024	0.000676	0.004826
hsa-miR-3132	139	260.955	0.08139359	0.02192138	0.91093622	7.572015783	0.000936	0.005292
hsa-miR-4430	259.105	482.99	0.10973452	0.0438913	0.90211755	8.46409558	0.002487	0.009713
hsa-miR-4294	626	1170.91	0.03388691	0.10738462	0.899638786	9.739423951	0.002186	0.008889
hsa-miR-3126-5p	186	346.72	0	0.08292271	0.895984108	7.987150865	0.000942	0.005292
hsa-miR-4669	216.285	401.66	0.07378875	0.01683001	0.894905481	8.202276077	0.000738	0.004936
hsa-miR-491-5p	88	162.645	0.04821183	0.08821173	0.884177886	6.900681731	0.00167	0.00776
hsa-miR-3180, hsa-miR-3180-3p	207.6	377.92	0.04359811	0.01900986	0.864829969	8.129391725	0.000259	0.003666
hsa-miR-4481	161.5	290.83	0.02189185	0.02032601	0.848665779	7.759550369	0.000112	0.003044
hsa-miR-5001-5p	86	154.205	0.01644434	0.05314593	0.841519079	6.846926755	0.000406	0.004034
hsa-miR-4257	180.5	322.345	0.10577221	0.01603546	0.84056048	7.912088807	0.002322	0.009236
hsa-miR-1292-5p	116	208.275	0.03657449	0.12524388	0.839167663	7.277082194	0.0041	0.012723
hsa-miR-30c-2-3p	91	162.87	0.03108162	0.12356026	0.834603378	6.92474781	0.003895	0.01238
hsa-miR-5703	98.5	176.26	0.02153625	0.15950622	0.830441572	7.037105302	0.007708	0.018443

hsa-miR-371b-5p	105	185.775	0.0673435	0.08149189	0.822405413	7.123810658	0.002393	0.009432
hsa-miR-4635	87	153.56	0.01625533	0.04706064	0.81901124	6.852353807	0.000339	0.003783
hsa-miR-3918	313.4	553.695	0.08754226	0.147361	0.815980744	8.697082356	0.009652	0.021836
hsa-miR-4532	284	500.345	0.06473513	0.09142239	0.815524473	8.555996317	0.00289	0.01016
hsa-miR-4665-5p	150	262.5	0	0.0134687	0.807289491	7.632463436	5.76E-05	0.003044
hsa-miR-4436a	89.5	154.5	0.00790063	0.05034417	0.786755044	6.877170786	0.000405	0.004034
hsa-miR-4251	85	145.83	0.03327561	0.01706793	0.779047189	6.798515058	0.000216	0.003616
hsa-miR-675-5p	128.5	220.325	0.01650833	0.03398734	0.777546396	7.394299448	0.000222	0.003621
hsa-miR-3138	302.04	515.91	0.06105597	0.05457733	0.77265044	8.623575246	0.001428	0.007147
hsa-miR-4468	85	144.84	0.03327561	0.02421465	0.769113296	6.793548112	0.000272	0.003666
hsa-miR-4722-5p	107.5	182.785	0.04604416	0.04475874	0.765853164	7.130354374	0.000774	0.005018
hsa-miR-583	96	163.5	0.02946278	0.10422797	0.764568629	6.966933662	0.003196	0.010707
hsa-miR-4726-5p	91	153.87	0.01554081	0.04521954	0.757122755	6.886268904	0.000394	0.004034
hsa-miR-4475	85.5	143.88	0.00827025	0.01847874	0.750771241	6.793213466	9.85E-05	0.003044
hsa-miR-4725-3p	685.57	1153.655	0.01561561	0.10805297	0.746700555	9.794422492	0.00351	0.011521
hsa-miR-4478	85	142.265	0	0.00263429	0.743043525	6.780912699	5.58E-05	0.003044
hsa-miR-4525	309.765	512.39	0.13803608	0.04523695	0.73223493	8.634242591	0.008304	0.019708
hsa-miR-4538	88	145.855	0.01607061	0.01110195	0.729008114	6.823842521	0.000104	0.003044
hsa-miR-1254	110.5	182.685	0.04479409	0.04400911	0.725336979	7.14984699	0.000863	0.005253
hsa-miR-1273e	117.25	192.97	0.05126147	0.02909482	0.719431656	7.232211561	0.000756	0.004951
hsa-miR-920	104.5	171.745	0.02029972	0.17428065	0.705874747	7.060147864	0.015656	0.030851
hsa-miR-4694-3p	79.5	129.4	0.06226097	0.01530061	0.704125893	6.663546419	0.000997	0.005532
hsa-miR-3944-5p	101.5	165.085	0.02089971	0.07782736	0.69969892	7.015027818	0.00185	0.008136

hsa-miR-198	134.835	218.51	0.06120025	0.03889718	0.697310528	7.422354329	0.001411	0.007147
hsa-miR-5787	6581.41	10626.475	0.02608858	0.10443832	0.687495258	13.02768309	0.004316	0.012963
hsa-miR-615-5p	162.335	261.2	0.04064007	0.02057432	0.686624064	7.685546365	0.000478	0.004262
hsa-miR-3158-5p	105.5	168.875	0.0201073	0.14341026	0.671402406	7.056654555	0.010594	0.023361
hsa-miR-627	97.5	154.775	0.02175713	0.04774199	0.666046537	6.940182829	0.000724	0.004936
hsa-miR-921	173.4	274.875	0.02772968	0.05595115	0.663819203	7.769592302	0.001116	0.005929
hsa-miR-4465	159.5	251.16	0.08423216	0.07792927	0.65542015	7.642559138	0.005926	0.015637
hsa-miR-215	90.5	141.82	0.03906667	0.00179494	0.648620814	6.823605622	0.000414	0.004035
hsa-miR-3925-5p	87.5	137.03	0.02424366	0.03065179	0.64700993	6.774504058	0.000417	0.004035
hsa-miR-3122	92.5	144.79	0.0076444	0.01465101	0.646380348	6.854550558	0.000131	0.003168
hsa-miR-4731-5p	92	143.415	0.07685943	0.00054235	0.64262384	6.842740089	0.00211	0.00875
hsa-miR-4654	101.5	158.21	0.04876598	0.06185676	0.639837947	6.984396655	0.002278	0.009188
hsa-miR-3150b-5p	109.5	170.535	0.04520317	0.0261638	0.639627405	7.093863411	0.00081	0.005091
hsa-miR-4708-3p	103	159.705	0.01373023	0.10081601	0.629157954	7.001011507	0.004807	0.013819
hsa-miR-711	196	302.135	0.04329225	0.00989975	0.624980614	7.926523853	0.000604	0.004538
hsa-miR-1224-5p	249.65	382.845	0.0829891	0.03721663	0.618842091	8.270695826	0.003712	0.011986
hsa-miR-4749-5p	2259.535	3440.08	0.13427467	0.01010893	0.612911676	11.4417337	0.011254	0.024489
hsa-miR-3622a-5p	92.5	141.465	0.0076444	0.01464548	0.6128636	6.837792183	0.000154	0.003278
hsa-miR-5196-5p	625.045	957.43	0.00915213	0.12397245	0.609672499	9.592622288	0.009166	0.02101
hsa-miR-6718-5p	90	136.785	0.03142697	0.01845503	0.604146582	6.793570078	0.00045	0.004181
hsa-miR-194-3p	91	138.335	0	0.01702147	0.604123261	6.809856271	0.000165	0.003423
hsa-miR-1273f	108	164.085	0.09166199	0.08692046	0.603718672	7.053710103	0.009821	0.022048
hsa-miR-5090	108.5	165.105	0.03258557	0.11901819	0.600944599	7.06164046	0.009357	0.021335

hsa-miR-5189	95	143.12	0.02977292	0.00582997	0.591533397	6.865302525	0.000349	0.003783
hsa-miR-4727-3p	107	161.465	0	0.08964466	0.590706403	7.036820188	0.004105	0.012723
hsa-miR-4773	79.5	119.75	0.04447212	0.09743016	0.588280677	6.606309611	0.006739	0.016771
hsa-miR-214-3p	107	160.035	0.15860339	0.08143205	0.587511298	7.026092335	0.026977	0.045714
hsa-miR-4516	2756	4136.75	0.07902354	0.11751639	0.583179497	11.71769409	0.014827	0.029688
hsa-miR-6724-5p	163.25	245	0.03248576	0.11544601	0.581256417	7.641186662	0.009526	0.021664

*CV: coefficient of variance, **AveExpr: Average expression level, *** adj. *p* value: adjust *p*-values
