# Supplementary material for: IGF-1-enhanced miR-513a-5p signaling desensitizes glioma cells to temozolomide by targeting the NEDD4L-inhibited Wnt/β-catenin pathway
Source: PLoS One. 2019 Dec 5;14(12):e0225913. doi: 10.1371/journal.pone.0225913 (PMC6894868; doi:10.1371/journal.pone.0225913)
Supplement: S3 Table — (PDF) [file pone.0225913.s005.pdf]

S3 Table. List of IGF-1-downregulated miRNAs in U87 MG cells (log2 (Ratio) ≦ -0.58; adj. *p* value ≦ 0.05)

Name	Normalized Intensity		CV*		log2 (Ratio)	AveExpr**	P.Value	adj. *p* value***
	Control	IGF-1	Control	IGF-1				
hsa-miR-4286	14391.82	1030.345	0.01849153	0.39787174	-3.86340803	11.88113407	0.001198	0.006247
hsa-miR-21-5p	6284.595	455.77	0.2485227	0.19337339	-3.776428643	10.70676203	0.000594	0.004502
hsa-miR-4324	2446.485	228.465	0.06157199	0.22250194	-3.437377346	9.536437397	0.000308	0.003783
hsa-miR-125b-5p	2685.735	250.92	0.00459954	0.07935648	-3.422284867	9.679951183	1.33E-05	0.002683
hsa-miR-1260a	9965.53	971.855	0.04079502	0.38325522	-3.412556675	11.57585199	0.001571	0.007519
hsa-let-7f-5p	3834.295	376.655	0.22952278	0.31878988	-3.366008733	10.20248599	0.00168	0.00776
hsa-miR-29a-3p	3618.5	365.47	0.1131449	0.06783365	-3.304596296	10.16424581	6.36E-05	0.003044
hsa-miR-4284	52844.005	5614.185	0.00505789	0.26454167	-3.260275033	14.05930545	0.000555	0.004486
hsa-let-7a-5p	4923.9	515.325	0.28350905	0.12987466	-3.232767769	10.61961322	0.000937	0.005292
hsa-let-7b-5p	2312.5	264.5	0.08469993	0.00267337	-3.125524163	9.609883416	2.11E-05	0.002683
hsa-miR-125a-5p	1685.385	189.75	0.27825953	0.05403451	-3.123478916	9.128641695	0.000782	0.005018
hsa-let-7c	2943.875	360.66	0.15654769	0.18692275	-3.032824745	9.998194628	0.00053	0.004438
hsa-miR-16-5p	1797.475	225.5	0.31115098	0.24772255	-2.981460238	9.285233799	0.002515	0.009736
hsa-miR-100-5p	1946.735	245.38	0.229752	0.11307715	-2.973299209	9.420896921	0.000658	0.004777
hsa-let-7d-5p	2296	319.805	0	0.16063322	-2.853225274	9.73829429	0.000181	0.003432
hsa-miR-363-5p	6831.135	956.84	0.58777103	0.32805782	-2.738884166	11.23167615	0.016716	0.031929
hsa-miR-99a-5p	1342.625	198.495	0.30691107	0.01072264	-2.72312379	8.994479282	0.001529	0.007441
hsa-miR-4443	12975.395	2131.83	0.23459372	0.31907255	-2.623173313	12.33177657	0.003651	0.011867
hsa-miR-221-3p	1581.095	259.04	0.20027227	0.03253827	-2.595445936	9.314372088	0.000493	0.004303

hsa-miR-1260b	24729.045	4204.69	0.01475202	0.28723265	-2.586444374	13.30061822	0.001459	0.007262
hsa-miR-27a-3p	1199.42	218.505	0.15160626	0.29982583	-2.481435069	8.979065791	0.002629	0.009961
hsa-let-7e-5p	1462.4	262.5	0.04796567	0.0134687	-2.477183797	9.27470008	1.10E-05	0.002683
hsa-miR-23a-3p	1152.975	231.705	0.11778214	0.19821146	-2.324290321	9.003979428	0.001013	0.005582
hsa-miR-222-3p	1196.63	242.365	0.20725758	0.21703494	-2.305254565	9.056472346	0.002305	0.009209
hsa-miR-4454	51765.09	11439.37	0.01161339	0.42026709	-2.244617359	14.53733454	0.00724	0.017714
hsa-miR-3135b	2641.385	563.075	0.1474428	0.1684649	-2.232321242	10.24303451	0.001045	0.005687
hsa-miR-23b-3p	973.92	207.7	0.10152971	0.07013192	-2.227350668	8.810256586	0.000177	0.003423
hsa-miR-29c-3p	1037.47	225.585	0.3128808	0.15682139	-2.174050265	8.895627446	0.004426	0.013155
hsa-miR-29b-3p	700.13	156.485	0.13507272	0.02245788	-2.155169824	8.367283544	0.000267	0.003666
hsa-miR-26a-5p	632.9	148.625	0.11641733	0.06170681	-2.086770568	8.257543626	0.000263	0.003666
hsa-miR-9-5p	408.31	96.73	0.07512501	0.04415305	-2.076294635	7.633335338	8.10E-05	0.003044
hsa-miR-4448	5942.25	1422.165	0.00053548	0.11817058	-2.06797456	11.5028062	0.000197	0.003461
hsa-miR-98-5p	800.125	192.535	0.04219884	0.03184157	-2.054827895	8.616025093	2.27E-05	0.002683
hsa-miR-92b-3p	409.59	101.375	0.0186794	0.09590844	-2.017678006	7.669071822	0.000122	0.003134
hsa-miR-27b-3p	621	159.2	0.09792461	0.0284264	-1.960577536	8.294693789	0.000151	0.003278
hsa-miR-181a-5p	559.11	143.785	0.2602501	0.05129272	-1.935316814	8.134478238	0.00273	0.010076
hsa-miR-22-3p	906.325	240.715	0.14928958	0.24672288	-1.926914886	8.85234363	0.003518	0.011521
hsa-miR-5100	10796	2873.995	0.06864097	0.22502224	-1.926163956	12.43342593	0.001919	0.008273
hsa-let-7g-5p	698.175	189.175	0.21304102	0.0008597	-1.86730921	8.497231973	0.001567	0.007519
hsa-miR-92a-3p	508.61	139.165	0.14567281	0.04232529	-1.862715225	8.05136381	0.000567	0.004486
hsa-miR-10a-5p	416.41	119.73	0.13785195	0.09295799	-1.794456723	7.797745833	0.000833	0.005181
hsa-miR-19b-3p	574	164.28	0.25377003	0.04726097	-1.782090645	8.250252314	0.003221	0.010709

hsa-miR-15a-5p	348.63	102.415	0.03095101	0.00807806	-1.766947629	7.5617335	1.29E-05	0.002683
hsa-miR-4632-5p	1063.455	328.305	0.0564579	0.1799078	-1.706269151	9.200258133	0.001422	0.007147
hsa-miR-3676-5p	1844.57	573.89	0.13997461	0.16441536	-1.687153286	9.998390697	0.002194	0.008889
hsa-miR-17-5p	480.75	150.105	0.02132719	0.05553972	-1.680264168	8.068846893	5.65E-05	0.003044
hsa-miR-34a-5p	526.875	167.075	0.15064814	0.07114449	-1.650560493	8.207804433	0.001084	0.005863
hsa-miR-20a-5p	459.5	146.795	0.07848193	0.04619472	-1.644807137	8.019292516	0.000188	0.003461
hsa-miR-99b-5p	453.675	144.135	0.1911647	0.03164283	-1.641297333	7.991564339	0.001739	0.007932
hsa-miR-138-5p	441.6	143.525	0.03714873	0.03473334	-1.621375455	7.975410723	4.31E-05	0.003044
hsa-miR-4701-5p	542.285	184.56	0.08531676	0.05792942	-1.553542613	8.303506033	0.000319	0.003783
hsa-miR-20b-5p	442.95	150.32	0.20768618	0.05343755	-1.544410202	8.003067597	0.00283	0.010102
hsa-miR-195-5p	426.06	148.335	0.07946363	0.06039736	-1.521233384	7.972015045	0.00031	0.003783
hsa-miR-24-3p	466.38	162.945	0.1898839	0.17926651	-1.515682691	8.094397702	0.005323	0.014718
hsa-miR-541-3p	438.245	153.62	0.20896393	0.04253135	-1.497100677	8.011119859	0.003058	0.01041
hsa-miR-4521	311.56	110.5	0.27942928	0.22397047	-1.485060678	7.512109745	0.014218	0.029138
hsa-miR-19a-3p	352.875	129.25	0.16080856	0.05744388	-1.440796452	7.733227562	0.001766	0.008012
hsa-miR-26b-5p	265	100.415	0.04802989	0.02232265	-1.399364803	7.34933364	7.49E-05	0.003044
hsa-miR-106a-5p	476	181.355	0.09507318	0.05829035	-1.390104092	8.19649823	0.000563	0.004486
hsa-miR-4695-5p	1236.655	472.475	0.11968133	0.01339458	-1.383013147	9.575536044	0.000716	0.004936
hsa-miR-15b-5p	356.93	136.8	0.06707936	0.05375666	-1.382991225	7.786377012	0.000274	0.003666
hsa-miR-3607-5p	387.44	151.665	0.03080725	0.02177291	-1.352913327	7.921030043	3.98E-05	0.003044
hsa-miR-30d-5p	246	97.5	0	0.02175713	-1.335354945	7.274837033	1.79E-05	0.002683
hsa-miR-3676-3p	350.825	141.135	0.10813448	0.06147448	-1.31081031	7.794972835	0.000925	0.005292
hsa-miR-4633-5p	392	155.825	0.25975351	0.03017654	-1.306500155	7.936704467	0.008395	0.019816

hsa-miR-193b-3p	210.335	84.75	0.21962719	0.02920205	-1.294100567	7.051884113	0.005295	0.014718
hsa-miR-30a-5p	231.875	94.5	0.1471392	0.00748261	-1.287130103	7.205787282	0.001611	0.007584
hsa-miR-3651	377.875	152.465	0.328876	0.07916776	-1.271590325	7.885865362	0.01906	0.035323
hsa-miR-766-3p	779.795	328.815	0.12369461	0.01731128	-1.240387232	8.981217825	0.001111	0.005929
hsa-miR-181b-5p	228.645	97.5	0.07821177	0.0652714	-1.228963832	7.220273993	0.000634	0.004679
hsa-miR-34b-5p	283.5	122.5	0.04240146	0.20203051	-1.224791916	7.534160225	0.005054	0.014239
hsa-miR-130a-3p	215.25	92.25	0.01478272	0.0268279	-1.222573236	7.138503987	3.75E-05	0.003044
hsa-miR-197-3p	553.395	238.735	0.12932223	0.08136312	-1.209233768	8.501491559	0.002156	0.00883
hsa-let-7i-5p	304.44	133.985	0.03047313	0.13177935	-1.190042164	7.654658029	0.001589	0.007523
hsa-miR-30e-5p	207.5	90.52	0.20787235	0.04327631	-1.181722778	7.090350268	0.006102	0.015818
hsa-miR-145-5p	212	93.5	0.10006228	0.00756264	-1.177426322	7.135586992	0.000694	0.004916
hsa-miR-4776-5p	624	277.52	0.0067991	0.06889654	-1.170651667	8.700059712	0.000255	0.003666
hsa-miR-1294	326.525	145.205	0.20561914	0.03121486	-1.154041341	7.758616478	0.006162	0.015927
hsa-miR-4649-5p	1037.335	480.495	0.02680957	0.04563498	-1.110780785	9.463016488	0.000153	0.003278
hsa-miR-30b-5p	200.125	91.855	0.18991625	0.02855986	-1.110637914	7.076311057	0.005465	0.014876
hsa-miR-3665	1418.655	671.91	0.09105902	0.11955073	-1.080360439	9.927131019	0.002884	0.01016
hsa-miR-4723-5p	1884.13	908.69	0.00515657	0.26276928	-1.077372548	10.34098693	0.014816	0.029688
hsa-miR-4763-5p	537.165	253.84	0.13646786	0.06217548	-1.076093049	8.524426503	0.002906	0.010166
hsa-miR-668	352.5	164.455	0.27080685	0.07270789	-1.074891955	7.897085836	0.017842	0.033564
hsa-miR-4323	536.25	255.54	0.11801596	0.05235368	-1.065304906	8.529068527	0.001939	0.008303
hsa-miR-10b-5p	193	93.085	0.05862025	0.01648409	-1.050837786	7.065797683	0.000256	0.003666
hsa-miR-106b-5p	190.5	92	0.02598293	0.03074377	-1.050182677	7.048312313	9.92E-05	0.003044
hsa-miR-6511b-5p	610.605	296.175	0.06625163	0.01539913	-1.04229023	8.73136564	0.000348	0.003783

hsa-miR-933	537.9	262.5	0.11804832	0.0134687	-1.030042271	8.551129317	0.001693	0.00776
hsa-miR-181c-5p	184.625	90.5	0.00287247	0.03906667	-1.02915592	7.013873175	1.00E-04	0.003044
hsa-miR-3607-3p	218.865	108	0.02671863	0.11785113	-1.023779257	7.261750299	0.00181	0.008125
hsa-miR-129-1-3p	181.835	90	0.13350002	0.03142697	-1.00853249	6.995763033	0.00273	0.010076
hsa-miR-423-3p	193.3	96.5	0.08559906	0.00732753	-0.999612575	7.092243959	0.000742	0.004936
hsa-miR-634	311.5	157.835	0.0385901	0.0586437	-0.981519145	7.791791467	0.000455	0.004181
hsa-miR-1234-5p	1336.69	680.47	0.13363556	0.1259859	-0.973339061	9.891309666	0.007024	0.017364
hsa-miR-4664-5p	193.335	98.335	0.11458689	0.00956376	-0.970607605	7.104903908	0.001845	0.008136
hsa-miR-129-2-3p	176.5	90	0.13220693	0	-0.96533947	6.974522831	0.002803	0.010102
hsa-miR-1248	263.125	135.235	0.01679589	0.07356818	-0.962132704	7.558436412	0.000586	0.004486
hsa-miR-4313	175	89.625	0.23435539	0.03747567	-0.945802359	6.958223771	0.015984	0.031146
hsa-miR-191-5p	188.5	98	0.03376107	0.01443075	-0.943374766	7.086322114	0.000115	0.003044
hsa-miR-4695-3p	279.57	144.41	0.18934082	0.02360124	-0.940192413	7.643922115	0.00874	0.020245
hsa-miR-151a-5p, hsa-miR-151b	188.5	98.665	0.02625861	0.00480172	-0.933713802	7.09131508	7.01E-05	0.003044
hsa-miR-30c-5p	171	89	0.17367535	0.01589004	-0.931248238	6.941266477	0.006941	0.017223
hsa-miR-21-3p	158	83	0.05370431	0.01703872	-0.927805045	6.838837236	0.00031	0.003783
hsa-miR-1229-3p	377	197.51	0.19131271	0.05527684	-0.920418573	8.084888284	0.01048	0.023194
hsa-miR-455-3p	159.5	84.25	0.05763253	0.01258944	-0.919666023	6.856380626	0.000356	0.003783
hsa-miR-93-5p	244.57	129.8	0.003296	0.05665571	-0.915111792	7.476543823	0.000329	0.003783
hsa-miR-107	197.5	105	0.02506201	0.0808122	-0.913596023	7.168684255	0.000937	0.005292
hsa-miR-449b-3p	215.5	115.58	0.07546847	0.18329226	-0.908959091	7.295007369	0.010702	0.023538
hsa-miR-28-5p	173	92.415	0.02452393	0.0369564	-0.904848914	6.98198682	0.000193	0.003461

hsa-miR-25-3p	176.665	95.215	0.05071204	0.02651233	-0.89108089	7.018403848	0.000364	0.003783
hsa-miR-3622a-3p	351.235	193.235	0.0009462	0.03626376	-0.862554327	8.025015318	0.000149	0.003278
hsa-miR-3620-3p	339.315	188.035	0.13622613	0.00725778	-0.844918513	7.977297671	0.004568	0.013351
hsa-miR-3176	325.235	183.375	0.11407718	0.04916489	-0.822848895	7.92920526	0.003776	0.012136
hsa-miR-1976	338	191.07	0.02928845	0.0153212	-0.822697181	7.989221389	0.000145	0.003278
hsa-miR-1538	164	93	0	0	-0.818393194	6.948355408	4.09E-05	0.003044
hsa-miR-4655-5p	1097.24	632.425	0.00159821	0.04153697	-0.795534442	9.701895264	0.00025	0.003666
hsa-miR-103a-3p	165	96.165	0.11142289	0.03183874	-0.774756177	6.974452383	0.003716	0.011986
hsa-miR-718	248.5	144.42	0.20203051	0.05023484	-0.769012381	7.557722179	0.019727	0.03625
hsa-miR-4534	451.5	264.5	0.13938539	0.00267337	-0.764419313	8.429330991	0.006532	0.016393
hsa-miR-6511a-5p, hsa-miR-6511b-5p	538.7	318.1	0.10422179	0.05890704	-0.757325951	8.690746898	0.004352	0.012983
hsa-miR-9-3p	141.5	84	0.00499722	0.01683588	-0.752434052	6.76843221	8.59E-05	0.003044
hsa-miR-1234-3p	173.335	103.55	0.04621986	0.04028904	-0.743050216	7.065123166	0.000755	0.004951
hsa-miR-4780	152.5	93	0.06955149	0	-0.711759781	6.895038702	0.00119	0.006242
hsa-miR-181d	143.5	88.875	0.05420331	0.02585763	-0.690381932	6.818655523	0.000898	0.005287
hsa-miR-1193	290.4	180.05	0.11979908	0.04885536	-0.685310254	7.834047529	0.007275	0.017749
hsa-miR-4687-5p	438.855	270.43	0.20670775	0.06421825	-0.684400183	8.419822538	0.030038	0.049739
hsa-miR-6511a-3p	260	163.22	0.04351426	0.1023273	-0.674797011	7.684286051	0.004989	0.014199
hsa-miR-3621	994.605	624.84	0.00086024	0.02704669	-0.670900494	9.62252936	0.000199	0.003461
hsa-miR-130b-3p	146.75	92.5	0.01204611	0.0076444	-0.665803971	6.864262369	0.000108	0.003044
hsa-miR-6131	328	207.575	0.00431163	0.05174492	-0.661022776	8.027033912	0.000723	0.004936
hsa-miR-4258	255.145	161.615	0.12668032	0.02157001	-0.653112689	7.662805811	0.008227	0.019578

hsa-miR-5739	932.965	592.345	0.10908649	0.0435835	-0.651765914	9.535491405	0.006439	0.01621
hsa-miR-4728-3p	294.1	187.44	0.03317944	0.07876862	-0.651722036	7.87390479	0.002685	0.009996
hsa-miR-374a-5p	139.5	88.25	0.1774103	0.0280439	-0.649438381	6.787959853	0.021037	0.038015
hsa-miR-361-5p	142	91	0.02987775	0.01554081	-0.641717555	6.828566304	0.000324	0.003783
hsa-miR-5010-5p	555.41	356.39	0.08298234	0.0077776	-0.637630204	8.796106331	0.002682	0.009996
hsa-miR-143-3p	127.5	82	0.0942809	0	-0.633588298	6.674346153	0.00382	0.012187
hsa-miR-4253	141.835	91.5	0.01161602	0.02318383	-0.632515152	6.83176353	0.000224	0.003621
hsa-miR-4713-5p	288.945	186.8	0.06879085	0.07419322	-0.629579768	7.858152415	0.004714	0.013641
hsa-miR-1290	1293.425	839.905	0.06169434	0.07729383	-0.623682249	10.02376546	0.004554	0.013351
hsa-miR-4758-3p	282.4	183.675	0.08313012	0.05139445	-0.619041329	7.82957882	0.004509	0.01329
hsa-miR-3180-5p	150.5	98	0.07047576	0.01443075	-0.617191311	6.923230387	0.002003	0.008428
hsa-miR-4723-3p	236.5	154.66	0.08072678	0.04882905	-0.611246243	7.577718971	0.004253	0.012817
hsa-miR-4446-3p	243.375	161.37	0.07772001	0.0872874	-0.593379927	7.628165295	0.00834	0.01974
hsa-miR-424-5p	146.25	97.125	0.0652714	0.06006312	-0.590286123	6.895611516	0.00399	0.012547
hsa-miR-125b-1-3p	136	90	0.14558081	0.03142697	-0.588281227	6.785637401	0.016646	0.031864
hsa-miR-320d	214	145.365	0	0.06143679	-0.55929349	7.461820242	0.001806	0.008125

*CV: coefficient of variance, **AveExpr: Average expression level, ***adj. *p* value: adjust *p*-values
