# Supplementary material for: IGF-1-enhanced miR-513a-5p signaling desensitizes glioma cells to temozolomide by targeting the NEDD4L-inhibited Wnt/β-catenin pathway
Source: PLoS One. 2019 Dec 5;14(12):e0225913. doi: 10.1371/journal.pone.0225913 (PMC6894868; doi:10.1371/journal.pone.0225913)
Supplement: Attachment — Submitted filename: Response to Reviewers.docx [file pone.0225913.s007.docx]

**PONE-D-19-20240
IGF-1-enhanced miR-513a-5p signaling desensitizes glioma cells to temozolomide by targeting the NEDD4L-inhibited Wnt/β-catenin pathway**

Dear Editor and Reviewers:

Thank you very much for reviewing our article and providing instructive comments. We have tried our best to address your concerns/suggestions in the revised manuscript, and hopefully the revised manuscript can reach the publishable level. The point-by-point responses are shown below.

**For Reviewer #1:**

**1. In the figure 1, the authors need to clearly demonstrate beta-catenin nuclear translocation upon IGF1 treatment. This could be done by immunofluorescence or by cell fractionation. Also showing beta-Catenin nuclear localisation is not enough to conclude to an increase of its activation upon IGF1. The authors need to assess its transcriptional activity upon IGF1 treatment by using a reporter gene containing TCF/LEF binding sites (top flash reporter gene).**

**Reply:** We tested the effects of IGF-1 on β-catenin nuclear accumulation. After cells were added with 200 ng/ml IGF-1 in the culture mediums for 48 h, the nuclear lysates were extracted for immunoblotting assays. As shown in figure below, IGF-1 treatment significantly enhanced β-catenin nuclear accumulation. Furthermore, we also conducted TCF reporter assays with IGF-1 treatment in TOPFlash reporter plasmids (M50 Super 8x TOPFlash, Addgene, Watertown, MA, USA)-transfected cells. We found IGF-1 treatment significantly enhanced luciferase activity, suggesting that IGF-1 induced β-catenin signaling activation. We added these results as the Fig. 1D and Fig. 1F. We also highlighted the modified sentences in the revised manuscript.

Fig. 1D. Effects of IGF-1 on β-catenin nuclear accumulation.

Fig. 1F. Effects of IGF-1 on β-catenin activation by using TCF reporter assays.

**2. The cell proliferation has been determined by MTT assay which reveals only 13% increase of cell proliferation upon IGF1. This is an indirect method to measure cell proliferation which need to be confirmed by counting viable cells following IGF1 treatment.**
**Reply:** Thank for your suggestion. We re-measured the effects of IGF-1 treatment on glioma cell growth. After cells were treated 200 ng/ml IGF-1 for 48h, cell viability was measured by manual cell counting with Trypan blue stain. As shown in figure below, we found IGF-1 increased the glioma cell growth ratio by 21% compared to the control group. We added this result as the Fig. 1B, and also highlighted the modified sentences in the revised manuscript.

Fig. 1B. Effects of IGF-1 treatment on glioma cell growth.

**3. Figure 1 shows an increase of cyclin D1 expression upon IGF1 treatment. Although cyclin D1 is a well known target of beta-Catenin many other factors may stimulate this gene upon growth factor stimulation. The authors have to show that this increase is beta-Catenin dependent by performing its functional invalidation using specific siRNA.**

**Reply:** Thank for your suggestion. We tested the β-catenin knockdown effects on IGF-1-enhanced cyclin D1 expression. First, we measured the knockdown effects of β-catenin shRNAs (sh-β-catenin) on endogenous β-catenin levels. We found sh-β-catenin significantly reduced endogenous β-catenin levels. Next, after cells were transfected with sh-β-catenin plasmids for 24 h, 200 ng/ml IGF-1 was added in the culture mediums for another 48 h. The β-catenin and cyclin D1 expression were measured by immunoblotting assays. As shown in figure below, inhibition of β-catenin significantly reduced IGF-1-enhanced cyclin D1 expression. We added this result as the Fig. 1G, and also highlighted the modified sentences in the revised manuscript.

Fig. 1G. Knockdown effects of sh-β-catenin on endogenous β-catenin levels.

Fig. 1G. Effects of knockdown β-catenin on IGF-1-enhanced cyclin D1 expression.

**4. Similar functional invalidation need to be conducted to assess whether or not the 13% increase of proliferation is due to WNT/beta-Catenin activation.**

**Reply:** Thank for your suggestion. We tested the knockdown effect of β-catenin on IGF-1-enhanced cell proliferation. After cells were transfected with sh-β-catenin plasmids for 24 h, 200 ng/ml IGF-1 was added in the culture mediums for another 48 h. Cell viability was measured by manual cell counting with Trypan blue stain. As shown in figure below, knockdown of β-catenin reduced IGF-1-induced cell growth, suggesting that β-catenin signaling involves in IGF-1 physiological functions. We added this result as the Fig. 1H, and also highlighted the modified sentences in the revised manuscript.

Fig. 1H. Knockdown effects of sh-β-catenin on IGF-1-induced cell growth.

**5. Cell sensitivity to TMZ mediated death has been carried out by using MTT assay. A direct method to measure cell death will be much more appreciated.**
**Reply:** Thank for your suggestion. We re-measured the effects of IGF-1 on TMZ sensitivity by manual cell counting with Trypan blue stain. As shown in figure below, IGF-1 treatment significantly attenuated TMZ cytotoxicity on glioma cells. We added this result as the Fig. 1J, and also highlighted the modified sentences in the revised manuscript.

Fig. 1J. Effects of IGF-1 treatment on TMZ cytotoxicity.

**6. Beta-Catenin invalidation need to be performed to confirm its contribution in TMZ resistance. This will also confirm the results obtained with GSK3-beta inhibition which is not functionally obvious in the case of M059K cells. The quantification of DNA damages would be also informative in this context.
Reply:** Thank for your suggestion. We tested the knockdown effect of β-catenin on IGF-1-insensitized TMZ cytotoxicity. After cells were transfected with sh-β-catenin plasmids for 24 h, 200 ng/ml IGF-1 and 200 μM TMZ were respectively added in the culture mediums for another 48 h. Cell viability was measured by manual cell counting with Trypan blue stain. DNA damage changes were measured by using OxiSelect™ Oxidative DNA Damage ELISA Kits (CELL BIOLABS, INC.; San Diego, CA, USA). As shown in figure below, knockdown of β-catenin attenuated IGF-1-insensitized TMZ cytotoxicity and DNA damage levels, suggesting that β-catenin signaling involves in regulating IGF-1 physiological functions. We added these results as the Fig. 1L and 1M. We also highlighted the modified sentences in the revised manuscript.

Fig. 1L. Knockdown effects of sh-β-catenin on IGF-1-insensitized TMZ cytotoxicity.

Fig. 1M. Knockdown effects of sh-β-catenin on IGF-1-insensitized TMZ-enhanced DNA damage.

**7. The authors show increased miR-513a-5p upon IGF1 stimulation. It would be interesting to assess the effect of miR-513a-5p invalidation (anti-miR) on GBM cell sensitivity to TMZ following IGF1 treatment.**

**Reply:** Thank for your suggestion. We measured the effects of miR-513a-5p inhibitor on IGF-1-insentizied TMZ cytotoxicity. After cells were transfected with 100nM miR-513a-5p inhibitors for 24 h, 200 ng/ml IGF-1 and 200 μM TMZ were respectively added in the culture mediums for another 48 h. The cell viability was measured by manual cell counting with Trypan blue stain. As shown in figure below, inhibition of miR-513a-5p significantly reduced IGF-1-insentizied TMZ cytotoxicity. We added this result as the Fig. 6F, and also highlighted the modified sentences in the revised manuscript.

Fig. 6F. Effects of miR-513a-5p inhibitors on IGF-1-insentizied TMZ cytotoxicity.

**8. The results clearly indicate that NEDD4L 3’UTR can be bound by miR-513a-5p. MiR-513a-5p overexpression induces the decrease of NEDD4L protein. These results are convincing but based on gene overexpression. In order to confirm the physiological relevance of these results, the authors need to perform miR-513a-5p invalidation (upon IGF1 treatment) and evaluate NEDD4L expression.**
**Reply:** Thank for your suggestion. We detected the effects of miR-513a-5p inhibitor on IGF-1-reduced NEDD4L expression. After cells were transfected with 100nM miR-513a-5p inhibitors for 24 h, 200 ng/ml IGF-1 were added in the culture mediums for another 48 h. The total cell lysates were extracted for immunoblotting assays. As shown in figure below, knockdown of miR-513a-5p significantly influenced IGF-1-reduced NEDD4L and –enhanced β-catenin expression, suggesting that miR-513a-5p involves in IGF-1-inhibited NEDD4L signaling. We added this result as the Fig. 5J, and also highlighted the modified sentences in the revised manuscript.

Fig. 5J. Effects of miR-513a-5p inhibitors on IGF-1-reduced NEDD4L expression.

**9. The consequences of miR-513a-5p invalidation on beta-Catenin nuclear localization need also to be assessed to confirm the relevance of this miR for activation of this pathway.**
**Reply:** We tested the effects of miR-513a-5p inhibitor on IGF-1-indcued β-catenin nuclear accumulation. After cells were transfected with 100nM miR-513a-5p inhibitors for 24 h, 200 ng/ml IGF-1 were added in the culture mediums for another 48 h. The nuclear lysates were extracted for immunoblotting assays. As shown in figure below, knockdown of miR-513a-5p significantly attenuated IGF-1-indcued β-catenin nuclear accumulation, suggesting that miR-513a-5p involves in IGF-1-mediated β-catenin signaling. We added this result as the Fig. 5I, and also highlighted the modified sentences in the revised manuscript.

Fig. 5I. Effects of miR-513a-5p inhibitors on IGF-1-indcued beta-catenin nuclear accumulation.

**10. To validate their study, the authors have to reproduce at least part of their data in patient-derived glioma stem cells which are definitely much more relevant to study GBM physiology than GBM cell lines.**

**Reply:** Thanks for your suggestion. Indeed, it is really a good idea to test the hypothesis of present study by using primary cells derived from GBM patients. However, it is difficult to collect GBM patient samples to test our hypothesis in a short time. Further, few GBM patients can be enrolled in these studies from our TMU university hospital every year. But we will keep this suggestion in mind when conducting future GBM studies.
