# Supplementary material for: IGF-1-enhanced miR-513a-5p signaling desensitizes glioma cells to temozolomide by targeting the NEDD4L-inhibited Wnt/β-catenin pathway
Source: PLoS One. 2019 Dec 5;14(12):e0225913. doi: 10.1371/journal.pone.0225913 (PMC6894868; doi:10.1371/journal.pone.0225913)
Supplement: Attachment — Submitted filename: Response to Reviewers.docx [file pone.0225913.s008.docx]

**PONE-D-19-20240R1
IGF-1-enhanced miR-513a-5p signaling desensitizes glioma cells to temozolomide by targeting the NEDD4L-inhibited Wnt/β-catenin pathway**

Dear Editor and Reviewers:

Thank you very much for reviewing our article and providing instructive comments. We have tried our best to address your concerns/suggestions in the revised manuscript, and hopefully the revised manuscript can reach the publishable level. The point-by-point responses are shown below.

**For the request:**

**As mentioned during the first review, the authors should provide a link for the microarray dataset. The whole miRNA microarray dataset should be deposited to a public database such as Gene Expression Omnibus (https://www.ncbi.nlm.nih.gov/geo/) and the GSE reference should be included in the material and method section.**

**Reply:** Thanks for the suggestion. We uploaded the array data to GEO database as GSE140297. All the information will be released on Nov 14, 2019. We add and highlight this information in the method section of revised manuscript.
